# A definition of the *Goniurosaurus yingdeensis* group (Squamata, Eublepharidae) with the description of a new species

**DOI:** 10.3897/zookeys.986.47989

**Published:** 2020-11-05

**Authors:** Shuo Qi, L. Lee Grismer, Zhi-Tong Lyu, Liang Zhang, Pi-Peng Li, Ying-Yong Wang

**Affiliations:** 1 State Key Laboratory of Biocontrol/ The Museum of Biology, School of Life Sciences, Sun Yat-sen University, Guangzhou, Guangdong 510275, China Sun Yat-sen University Guangzhou China; 2 Institute of Herpetology, Shenyang Normal University, Shenyang, Liaoning 110034, China Shenyang Normal University Shenyang China; 3 Herpetology Laboratory, Department of Biology, La Sierra University, Riverside, California 92515, USA La Sierra University Riverside United States of America; 4 Guangdong Key Laboratory of Animal Conservation and Resource Utilization/ Guangdong Public Laboratory of Wild Animal Conservation and Utilization/ Guangdong Institute of Applied Biological Resources, Guangdong Academy of Science, Guangzhou, Guangdong 510275, China Guangdong Academy of Science Guangzhou China

**Keywords:** *Goniurosaurus varius* sp. nov., *Goniurosaurus zhelongi*, morphology, phylogeny, taxonomy

## Abstract

A definition of the *Goniurosaurus yingdeensis* group is presented in this study, on the basis of morphological and phylogenetic analyses based on a series of additional specimens. Moreover, a new species of this group, *Goniurosaurus varius***sp. nov.**, is proposed for northern Guangdong Province, China. The new species can be distinguished from the other two congeners of this group by the following unique characters: one or two internasals; enlarged supraorbital tubercles absent; paravertebral tubercles between limb insertions 27–29; dorsal tubercle rows at midbody 21–24; ten precloacal pores in males and absent in females; body bands with black spots; iris orange-red.

## Introduction

The eublepharid genus *Goniurosaurus* Barbour, 1908, currently contains 21 species that are distributed in east and Southeast Asia (Uetz et al. 2020). Nine of those species were described in last decade (Wang et al. 2014; Yang and Chan 2015; Zhou et al. 2018; Zhou et al. 2019; Zhu et al. 2020). In previous studies based on morphological analysis, the genus *Goniurosaurus* was suggested to be divided into three species groups (Grismer et al. 1999, 2002; Wang et al. 2013, 2014). The *G. kuroiwae* group is composed of five endemic species in the Ryukyu Archipelago, Japan: *G. kuroiwae* (Namiye, 1912), *G. orientalis* (Maki, 1931), *G. splendens* (Nakamura & Uéno, 1959), *G. toyamai* Grismer, Ota & Tanaka, 1994, and *G. yamashinae* (Okada, 1936). The *G. lichtenfelderi* group is composed of four species in the Gulf of Tonkin, Hainan island and Guangdong Province, China: *G. hainanensis* Barbour, 1908, *G. lichtenfelderi* (Mocquard, 1897), *G. yingdeensis* Wang, Yang & Cui, 2010, and *G. zhelongi* Wang, Jin, Li & Grismer, 2014. The *G. luii* group is composed of five species from northern Vietnam, through the China-Vietnam border, to southern Guangxi Zhuang Autonomous Region and Guizhou Province, China, include *G. araneus* Grismer, Viets & Boyle, 1999, *G. bawanglingensis* Grismer, Shi, Orlov, & Ananjeva, 2002, *G. catbaensis* Ziegler, Nguyen, Schmitz, Stenke & Rösler, 2008, *G. huuliensis* Orlov, Ryabov, Nguyen, Nguyen & Ho, 2008, *G. liboensis* Wang, Yang & Grismer, 2013, and *G. luii* Grismer, Viets & Boyle, 1999. However, a recent molecular phylogenetic study suggested that the genus *Goniurosaurus* could be divided into four species groups (Liang et al. 2018). According to their proposition, *G. yingdeensis* and *G. zhelongi* formed an independent clade, the *G. yingdeensis* group, which only occurs in northern Guangdong Province, China. However, the morphological definition of this newly proposed species group has not been given so far, which may bring chaos to subsequent research.

During the herpetological surveys conducted from 2015 to 2019, a number of *Goniurosaurus* specimens were collected from northern Guangdong Province, China that should be placed in the *G. yingdeensis* group on the basis of both morphological and molecular analyses. Furthermore, these specimens can be distinguished from congeners by discrete morphological differences and genetic divergences, and represent an unidentified taxon within the *G. yingdeensis* group. In the present study, this taxon is described as a new species and the *Goniurosaurus yingdeensis* group is revised and defined.

## Materials and methods

### Sampling

Sixteen specimens of *Goniurosaurus yingdeensis* were collected from the Shimentai National Nature Reserve, Yingde City, Guangdong Province (including six type specimens) for morphological comparison, and four specimens (SYS r001271, 1272, 1493, 2115) were used in the phylogenetic analysis. Nine specimens of *G. zhelongi* were collected from the Shimentai National Nature Reserve, Yingde City, Guangdong Province (including five type specimens) for morphological comparison, and four specimens (SYS r000816, 1491, 1492, 2108) were used in the phylogenetic analysis. Five specimens of the undescribed species were collected from the Nanling National Nature Reserve, Chengjia Yao Ethnic Township, Yangshan County, Guangdong Province, China, and all of them were used in phylogenetic analysis. Following euthanasia, all specimens were fixed in 10% formalin and transferred to 75% alcohol; they are deposited in the Museum of Biology, Sun Yat-sen University (**SYS**), Guangdong Province, China.

Due to the cryptic diversity in genus *Goniurosaurus*, we choose sequences from type series or topotype specimen for molecular analysis if available, to ensure the taxonomic identity of the species being studied. A total of 10 samples from four known species (one sample of *Goniurosaurus bawanglingensis*, four samples of *G. yingdeensis*, three samples of *G. zhelongi*, and two samples of *G. zhoui*) and five samples of the unidentified species were used. Tissues samples were taken before the specimens were fixed in 10% formalin, preserved in 99% alcohol, and stored at –40 °C. Sequences of other species of *Goniurosaurus* follow Liang et al. (2018); for details see Table 1.

**Table 1.** Localities, voucher information, and GenBank accession numbers for all specimens used in this study.

Species name	Locality	Specimen voucher	16S	Cytb	References
**Ingroup**: *Goniurosaurus*					
(1) *G. varius* sp. nov.	Yangshan, Guangdong, China	SYS r002330	MT995753	MT995768	This study
(2) *G. varius* sp. nov.		SYS r002331	MT995754	MT995769	This study
(3) *G. varius* sp. nov.		SYS r002333	MT995755	MT995770	This study
(4) *G. varius* sp. nov.		SYS r002362	MT995756	MT995771	This study
(5) *G. varius* sp. nov.		SYS r002363	MT995757	MT995772	This study
(6) *G. bawanglingensis*	Bawangling, Hainan, China	SYS r002162	MT995758	MT995773	This study
(7) *G. bawanglingensis*		BL-RBZ-021	MH247190	MH247201	Liang et al. 2018
(8) *G. hainanensis*	Jianfengling, Hainan, China	SYS r000349	KC765080	N/A	Wang et al., 2013
(9) *G. huuliensis*	Vietnam	N/A	AB853453	AB853479	Honda et al. 2014
(10) *G. kuroiwae*	Northern Okinawajima Island, Japan	N/A	AB853448	AB853473	Honda et al. 2014
(11) *G. liboensis*	Libo, Guizhou, China	SYS r000217	KC900230	N/A	Wang et al. 2013
(12) *G. luii*	Jingxi, Guangxi, China	SYS r000255	KC765083	N/A	Wang et al. 2013
(13) *G. luii*		SYS r000256	KC765084	N/A	Wang et al. 2013
(14) *G. luii*	Cao Bang,Vietnam	ZFMK 87057	EU499391	N/A	Ziegler et al. 2008
(15) *G. orientalis*	Iejima Island, Japan	N/A	AB853446	AB853467	Honda et al. 2014
(16) *G. splendens*	Tokunoshima Island, Japan	N/A	AB853451	AB853477	Honda et al. 2014
(17) *G. yamashinae*	Kumejima Island, Japan	N/A	AB853442	AB853460	Honda et al. 2014
(18) *G. yingdeensis*	Yingde, Guangdong, China	SYS r001271	MT995759	MT995774	This study
(19) *G. yingdeensis*		SYS r001272	MT995760	MT995775	This study
(20) *G. yingdeensis*		SYS r001493	MT995761	MT995776	This study
(21) *G. yingdeensis*		SYS r002115	MT995762	MT995777	This study
(22) *G. zhelongi*	Yingde, Guangdong, China	SYS r000816	KJ423105	MT995778	Wang et al. 2014, this study
(23) *G. zhelongi*		SYS r001491	MT995763	MT995779	This study
(24) *G. zhelongi*	Yingde, Guangdong, China	SYS r001492	MT995764	MT995780	This study
(25) *G. zhelongi*		SYS r002108	MT995765	MT995781	This study
(26) *G. zhoui*	Central area, Hainan, China	SYS r002213	MT995766	MT995782	This study
(27) *G. zhoui*		SYS r002214	MT995767	MT995783	This study
(28) *G. zhoui*		BL-RBZ-001	MH247196	MH247207	Liang et al. 2018
**Outgroup**					
(29) *Hemitheconyx taylori*	East Africa	N/A	AB308457	N/A	Jonniaux and Kumazawa 2008

### Species delimitation

The general lineage concept (GLC; de Queiroz 2007) adopted herein proposes that a species constitutes a population of organisms evolving independently from other such populations owing to a lack of gene flow. By “independently”, it is meant that new mutations arising in one species cannot spread readily into another species (Barraclough et al. 2003; de Queiroz 2007). Integrative studies on the nature and origins of species are using an increasingly wide range of empirical data to delimit species boundaries (Coyne and Orr 1998; Fontaneto et al. 2007; Knowles and Carstens 2007; Leaché et al. 2009), rather than relying solely on morphology and traditional taxonomic methods. Under the GLC implemented herein, molecular phylogenies were used to recover monophyletic mitochondrial lineages of individuals (populations) in order to develop initial species-level hypotheses – the grouping stage of Hillis (2019). Discrete color pattern data and univariate and multivariate analyses of morphological data were then used to search for characters and morphospatial patterns bearing statistically significant differences that were consistent with the previous designations of the species-level hypotheses, the construction of boundaries representing the hypothesis-testing step of Hillis (2019), thus providing independent diagnoses to complement the molecular analyses.

### Morphological characters

Measurements were taken following Ziegler et al. (2008) using digital calipers (Neiko 01407A Stainless Steel 6-Inch Digital Caliper, USA) to the nearest 0.1 mm. Abbreviations of morphological characters are as follows: **SVL** snout-vent length (from tip of snout to vent); **TaL** tail length (from vent to tip of tail); **HL** head length (from tip of snout to posterior margin of ear opening); **HW** maximum head width; **SE** snout-to-eye distance (measured from tip of snout to the boney anterior margin of the orbit); **EE** eye-to-ear distance (from the boney posterior margin of the orbit to posterior margin of ear opening); **SPL** supralabials; **IFL** infralabials; **N** nasal scales surrounding nare; **IN** internasals; **PostIN** granular scales bordering the internasals; **PM** postmentals; **GP** gular scales bordering postmentals; **CIL** eyelid fringe scales or ciliaria; **PO** preorbital scales (number of scales in a line from posterior margin of external naris to anterior margin of the orbit); **GST** granular scales surrounding dorsal tubercles; **PTL** paravertebral tubercles between limb insertions; **DTR** dorsal tubercle rows at midbody; **MB** scales around midbody; **PP** precloacal pores; **PAT** postcloacal tubercles. Bilateral scale counts are given as left/right.

Data of characters of known congeners were taken from the literature (Grismer et al. 1999, 2002; Orlov et al. 2008; Ziegler et al. 2008; Blair et al. 2009; Wang et al. 2010, 2013, 2014; Yang and Chan 2015; Zhou et al. 2018) and 30 museum specimens of the seven species listed in the Appendix 1 were examined.

### DNA Extraction, Polymerase Chain Reaction (PCR), and sequencing

Genomic DNA was extracted from muscle tissue samples, using a DNA extraction kit from Tiangen Biotech (Beijing) Co., Ltd. Partial segments of the mitochondrion genes 16S ribosomal RNA gene (16S) and Cytochrome b gene (Cytb) were amplified. Primers used for 16S were r16S-5L (5’- GGTMMYGCCTGCCCAGTG -3’) and 16sbr-H (5’- CCGGTCTGAACTCAGATCACGT-3’) (Palumbi et al. 1991) and for Cytb the primers were L14731 (5’- TGGTCTGAAAAACCATTGTTG-3’) (Honda et al. 2014) and H15149m (5’- GCMCCTCAGAAKGATATTTGYCCTCA-3’) (Chambers and MacAvoy 1999). The PCR procedure was performed with an initial denaturation at 94 °C for 5 min, 35 cycles of 94 °C for 30 s, 55 °C for 30 s and 72 °C for 1 min, followed by a final extension at 72 °C for 10 min (Liang et al. 2018). PCR products were purified with spin columns and then sequenced with forward primers using BigDye Terminator Cycle Sequencing Kit as per the guidelines on an ABI Prism 3730 automated DNA sequencer by Shanghai Majorbio Bio-pharm Technology Co., Ltd.

### Phylogenetic analyses

Twenty sequences from eleven known *Goniurosaurus* species and one out-group sequence from *Hemitheconyx taylori* in the Eublepharidae used to root the tree, were obtained from GenBank and incorporated into our dataset (Table 1). DNA sequences were aligned by the Clustal W with default parameters (Thompson et al. 1997) and trimmed with gaps partially deleted in MEGA 6 (Tamura et al. 2013). Two gene segments, with 482 base pairs (bp) of 16S and 396 bp of Cytb, were concatenated seriatim into an 878 bp sequence, and further divided into two partitions based upon each gene. The partitions were tested in jmodeltest v2.1.2 with Akaike and Bayesian information criteria, all resulting the best-fitting nucleotide substitution models of GTR+I+G. Sequence data were analyzed using Bayesian inference (BI) in MrBayes 3.2.4 (Ronquist et al. 2012), and maximum likelihood (ML) in RaxmlGUI 1.3 (Silvestro and Michalak 2012). Two independent runs were conducted in the BI analysis with 10,000,000 generations each and sampled every 1000 generations with the first 25% of samples discarded as burn-in, resulting in a potential scale reduction factor (PSRF) of < 0.005. In the ML analysis, a bootstrap consensus tree inferred from 1000 replicates was generated. Uncorrected pairwise sequence divergences utilizing the 16s gene were calculated using MEGA 6 (provide ref for MEGA 6).

### Statistical analyses of morphology

An analysis of variance (ANOVA) was conducted on characters with statistically similar variances (i.e., *p* values ≤ 0.05 in a Levene’s test) to search for the presence of statistically significant mean differences (*p* < 0.05) across the data set. Characters bearing statistical differences were subjected to a TukeyHSD test to ascertain which population pairs differed significantly from each other for those particular characters. The mensural characters were scaled to SVL in order to remove any potential effects of allometry using the following equation: X_adj_ = log(X)-β[log(SVL) – log(SVL_mean_)], where X_adj_ = adjusted value; X = measured value; β = unstandardized regression coefficient for each population; and SVL_mean_ = overall average SVL of all populations (Thorpe 1975, 1983; Turan 1999; Lleonart et al. 2000). Boxplots were generated in order to visualize the range, mean, and degree of differences between pairs of species bearing statistically different mean values for sets of characters.

## Results

**Figure 1.**
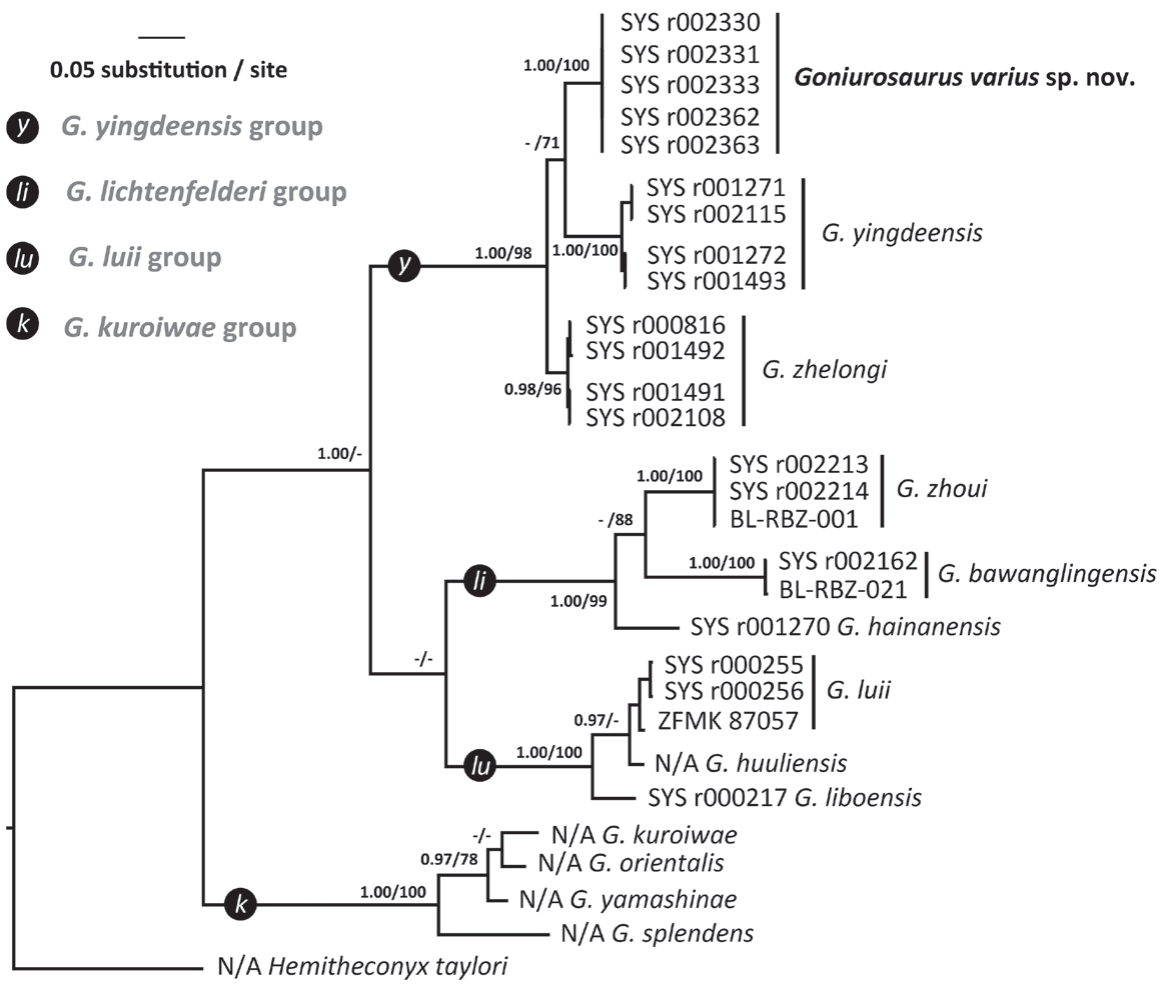
Bayesian inference tree of 13 species of *Goniurosaurus*, based on the partial DNA sequences of the mitochondrial 16S rRNA and Cytb genes. *Hemitheconyx taylori* is the outgroup. Numbers before slash indicate Bayesian posterior probabilities (> 0.94 retained) and numbers after slash are bootstrap support for ML (1000 replicates) analyses (> 70 retained). The hyphen represents bootstrap values ≤ 0.94 or ≤ 70.

The ML and BI analyses resulted in essentially identical topologies (Fig. 1). Uncorrected pairwise sequence divergences are reported in Table 2. The phylogenetic analyses showed that *Goniurosaurus* can be divided into four strongly supported clades consistent with the recognition of the four species groups proposed by Liang et al. (2018), i.e., the *G. kuroiwae* group, *G. lichtenfelderi* group, *G. luii* group, and *G. yingdeensis* group.

**Table 2.** Uncorrected *P*-distance of 16S gene among 13 *Goniurosaurus* species and the outgroup species used in this study.

		1	2	3	4	5	6	7	8	9	10	11	12	13	14
**1**	*G. varius* sp. nov.	0–0.3													
**2**	*G. zhelongi*	3.3–3.9	0–0.3												
**3**	*G. yingdeensis*	4.1–4.7	4.7	0–0.5											
**4**	*G. huuliensis*	11.1	13.3	13.6–13.9	/										
**5**	*G. luii*	12.3–12.6	13.6–13.9	12.6–13.3	1.6–1.9	0–0.8									
**6**	*G. liboensis*	12.7	12.6–12.7	13.0–13.3	3.9	3.6–4.2	/								
**7**	*G. zhoui*	14.8–15.1	16.7–16.8	17.1	14.1	14.7–15.3	14.6	0–0							
**8**	*G. hainanensis*	15.1–15.4	17.0	16.4	13.4	13.7–14.1	13.7	5.6	/						
**9**	*G. bawanglingensis*	16.2–16.8	15.8–16.5	17.9–18.2	15.4–15.7	14.7–15.7	15.0–15.3	5.6–5.8	74–7.7	0–0.3					
**10**	*G. orientalis*	15.7–16.0	17.1–17.4	15.4–15.7	19.0	19.3–19.7	19.3	18.0	18.8	19.1–19.5	/				
**11**	*G. yamashinae*	16.0–16.3	17.7–18.0	16.0–16.3	18.6	19.3–19.6	19.3	17.1	18.4	19.7–20.1	1.1	/			
**12**	*G. kuroiwae*	17.4–17.7	18.8–19.1	16.8–17.1	18.7	18.7–19.1	19.0	17.0	18.1	18.4–18.7	1.4	1.9	/		
**13**	*G. splendens*	17.8–18.1	19.5–19.9	16.8–17.2	20.4	19.7–20.1	20.0	18.8	18.8	21.0–21.4	4.5	4.2	5.0	/	
**14**	*Hemitheconyx taylori*	17.4–17.7	18.0–18.4	19.3–19.4	23.1	24.5–24.6	23.1	22.2	24.0	25.8–26.2	18.5	18.1	19.2	20.4	/

**Figure 2.**
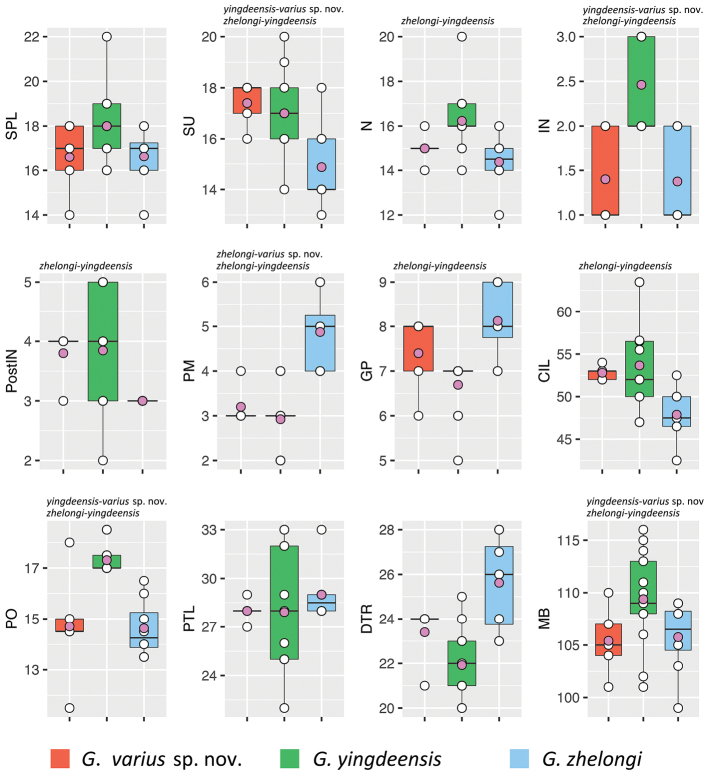
Boxplots showing characters bearing significantly different mean values. Species pairs bearing significantly different mean values between them are listed above each plot. Abbreviations are in the materials and methods.

**Figure 3.**
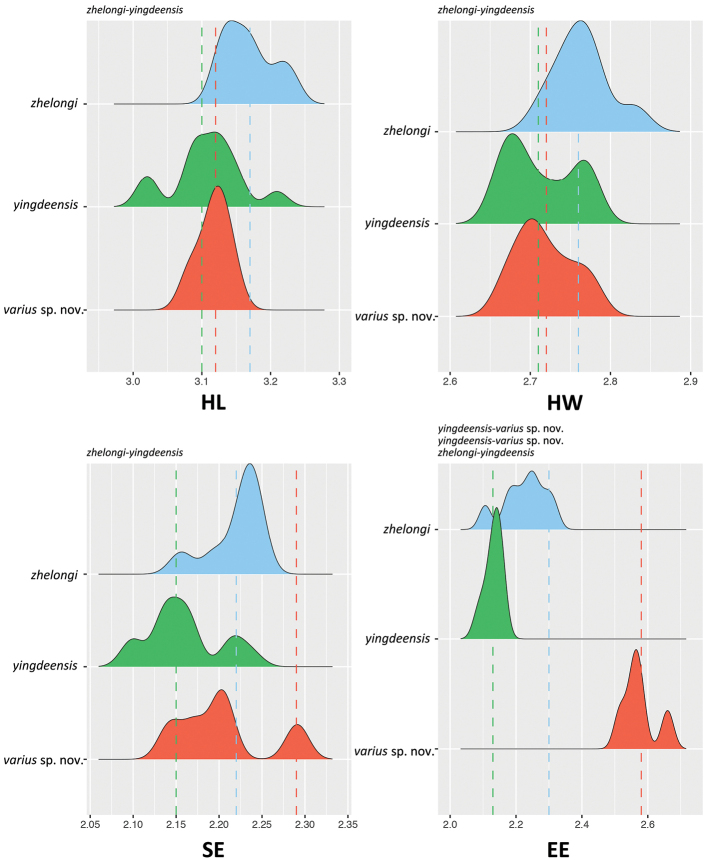
Ridge plots showing characters bearing significantly different mean values. Species pairs bearing significantly different mean values between them are listed above each plot. Abbreviations are in the Materials and methods.

The *Goniurosaurus yingdeensis* group is divided into three subclades with moderate genetic differences among them (3.3–4.7%), two of which represent *G. yingdeensis* and *G. zhelongi*, respectively; the third subclade is composed of the new population from northern Guangdong Province with a high nodal support value (1.00 in BI and 100% in ML) and low intrapopulational genetic differentiation (0–0.3%) and represents an unnamed species of *Goniurosaurus* (Table 2). Additionally, this population has a combination of characteristics (see below) distinguishing it from other species in the *G. yingdeensis* group while showing significant differences from all known congeners. ANOVAs and subsequent TukeyHSD tests recovered significantly different mean values among various combinations of species across various combinations of characters (Tables 3, 4; Figs 2, 3).

**Table 3.** ANOVA*F* values and TukeyHSD adjusted *p* values for pairs of species bearing statistically significant mean vales in the listed characters.

	ANOVA F	TukeyHSD *p* adjusted
**Eye to ear distance (EE)**	169.5	
*yingdeensis-varius*		2.24E-14
*zhelongi-varius*		7.50E-12
*zhelongi-yingdeensis*		0.0004
**Snout to eye distance (SE)**	5.717	
*zhelongi-yingdeensis*		0.0098
**Head length (HL)**	5.087	
*zhelongi-yingdeensis*		0.0126
**Maximum head width (HW)**	4.292	
*zhelongi-yingdeensis*		0.0244
**Infralabials (IFL)**	6.493	
*zhelongi-varius*		0.0168
*zhelongi-yingdeensis*		0.0106
**Nasal scales surrounding nares (N)**	5.773	
*zhelongi-yingdeensis*		0.0086
**Internasals (IN)**	13.75	
*yingdeensis-varius*		0.0022
*zhelongi-yingdeensis*		0.0003
**Granular scales bordering internasals (PostIN)**	3.548	
*zhelongi-yingdeensis*		0.0449
**Postmentals (PM)**	21.43	
*zhelongi-varius*		0.0007
*zhelongi-yingdeensis*		4.58E-06
**Gular scales bordering postmentals (GP)**	9.196	
*zhelongi-yingdeensis*		0.0008
**Eyelid fringe scales or ciliaria (CIL)**	4.898	
*zhelongi-yingdeensis*		0.0146
**Preorbital scales (PO)**	15.52	
*yingdeensis-varius*		0.0012
*zhelongi-yingdeensis*		0.0001
**Dorsal tubercle rows at midbody (DTR)**	12.2	
*zhelongi-yingdeensis*		0.0001

**Table 4.** Summary statistics for meristic and adjusted mensural characters among the species of the *Goniurosaurs yingdeensis* group. SD = standard deviation and N = sample size.

Scaled mensural characters	*varius* sp. nov. (N = 5)	*yingdeensis* (N = 13)	*zhelongi* (N = 8)
HL			
**mean** (±**SD)**	3.1 (±0.02)	3.1 (±0.05)	3.2 (±0.04)
**Range**	3.08–3.14	3.02–3.21	3.12–3.23
HW			
**Mean** (±**SD)**	2.7 (±0.04)	2.7 (±0.04)	2.8 (±0.03)
**Range**	2.67–2.77	2.66–2.77	2.71–2.83
SE			
**Mean** (±**SD)**	2.2 (±0.06)	2.2 (±0.04)	2.2 (±0.03)
**Range**	2.14–2.29	2.10–2.24	2.16–2.24
EE			
**Mean** (±**SD)**	2.6 (±0.05)	2.2 (±0.02)	2.2 (±0.07)
**Range**	2.52–2.66	2.01–2.15	2.11–2.31
Meristic characters			
SPL			
**Mean** (±**SD)**	16.6 (±1.67)	18.0 (±1.58)	16.6 (±1.30)
**Range**	14–18	16–22	14–18
IFL			
**Mean** (±**SD)**	17.4 (±0.89)	17.0 (±1.53)	14.9 (±1.64)
**Range**	16–18	14–20	13–18
N			
**Mean** (±**SD)**	15 (±0.71)	16.2 (±1.42)	14.4 (±1.19)
**Range**	14–16	14–20	12–16
IN			
**Mean** (±**SD)**	1.4 (±0.54)	2.5 (±0.52)	1.4 (±0.52)
**Range**	1 or 2	2 or 3	1 or 2
PostIN			
**Mean** (±**SD)**	3.8 (±0.44)	3.8 (±0.99)	3 (±0.00)
**Range**	3 or 4	2–5	3
PM			
**Mean** (±**SD)**	3.2 (±0.44)	2.9 (±0.64)	4.9 (±0.83)
**Range**	3 or 4	2–4	4–6
GP			
**Mean** (±**SD)**	7.4 (±0.89)	6.7 (±0.63)	8.1 (±0.83)
**Range**	6–8	5–7	7–9
CIL			
**Mean** (±**SD)**	52.8 (±0.83)	53.7 (±5.33)	47.9 (±3.01)
**Range**	52–54	47–63.5	42.5–52.5
PO			
**Mean** (±**SD)**	14.7 (±2.31)	17.3 (±0.56)	14.6 (±1.13)
**Range**	11.5–18	17–18.5	13.5–16.5
PTL			
**Mean** (±**SD)**	28.0 (±0.71)	27.9 (±3.64)	29.0 (±1.69)
**Range**	27–29	22–33	28–33
DTR			
**Mean** (±**SD)**	23.4 (±1.34)	21.9 (±1.50)	25.6 (±2.07)
**Range**	21–24	20–25	23–28
MB			
**Mean** (±**SD)**	105.4 (±3.36)	109.4 (±4.59)	105.8 (±3.49)
**Range**	101–110	101–116	99–109

Based on phylogeny and corroborating statistically significant differences in morphology (Figs 1–3), we propose that the northern Guangdong Province population is a new species of the *Goniurosaurus yingdeensis* group. The discovery of this new species has provided valuable new morphological and genetic information on this species group. The previous designation of the *Goniurosaurus yingdeensis* species group was on the based solely on molecular data and lacked a morphological definition. Therefore, along with the description of a new species, we provide the first morphological definition of the *Goniurosaurus yingdeensis* group.

### Systematics

**Class Reptilia Laurenti, 1768**

**Order Squamata Oppel, 1811**

**Family Eublepharidae Boulenger, 1883**

**Genus *Goniurosaurus* Barbour, 1908**

#### *Goniurosaurus yingdeensis* group

**Morphological definition.** This species group can be differentiated from the other species groups by the combination of the following characters: (1) base of claws sheathed by four scales, two lateral scales of claw short and shell-shaped; (2) precloacal pores fewer than 15 in males and absent in most females; precloacal pores form a continuous transverse series not extending onto the femora; (3) enlarged row of supraorbital tubercles indistinct or absent; (4) nuchal loop rounded posteriorly; and (5) four body bands between the nuchal loop and the caudal constriction.

**Comparison.** The *Goniurosaurus yingdeensis* group can be distinguished from the three other known species groups by the base of claws being sheathed by four scales, two lateral scales of claw short and shell-shaped vs. claws sheathed by four scales, two lateral scales of claw long, curved in *G. lichtenfelderi* group and *G. luii* group, and not sheathed by four scales in *G. kuroiwae* group; precloacal pores less than 15 in males vs. 17–46 in *G. lichtenfelderi* group (37–46 in *G. bawanglingensis*, 24–32 in *G. hainanensis*, 17–32 in *G. lichtenfelderi*, 36–38 in *G. zhoui*), 16–33 in *G. luii* group (18–22 in *G. araneus*, 16–21 in *G. catbaensis*, 25–28 in *G. huuliensis*, 26–28 in *G. kadoorieorum*, 31–33 in *G. kwangsiensis*, 23–28 in *G. liboensis*, 23–29 in *G. luii*) and absent in *G. kuroiwae* group.

**Figure 4.**
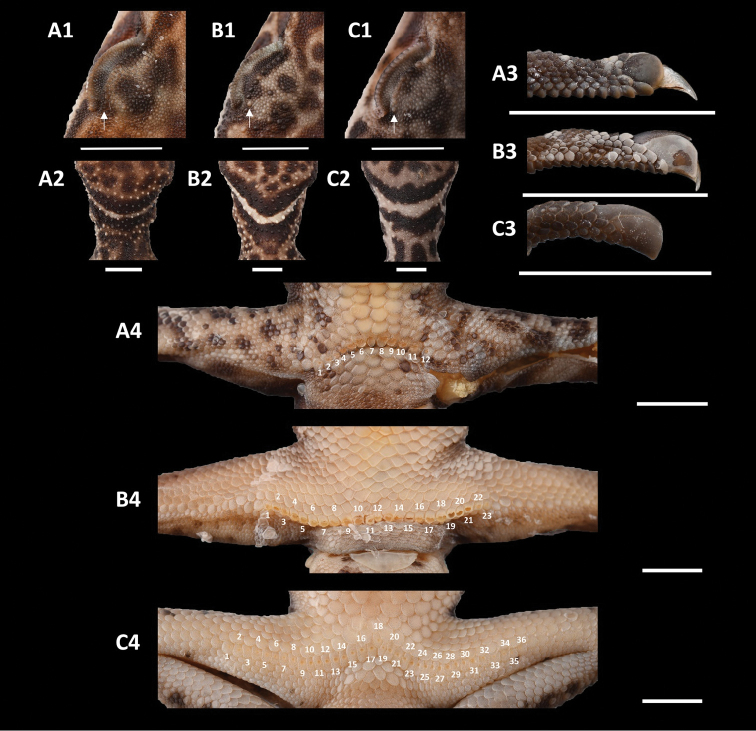
Comparisons of morphological characteristics of three species group in the genus *Goniurosaurus***A***Goniurosaurus yingdeensis* group (*G. yingdeensis*) **B***Goniurosaurus luii* group (*G. liboensis*) **C***Goniurosaurus lichtenfelderi* group (*G. zhoui*) **1** enlarged row of supraorbital tubercles **2** shape of nuchal loop **3** sheathed claws **4** number and position of precloacal pores. Scale bars: 5 mm. Photographs by Shuo Qi.

Summary statistics of the species of the *Goniurosaurus yingdeensis* group are listed in Table 4. Additional comparisons of morphological characteristics are provided in Table 5 and Fig. 4.

**Table 5.** Diagnostic characters distinguishing *Goniurosaurus varius* sp. nov. from all other known species of *Goniurosaurus*. Data come from Grismer et al. 1999, 2002; Orlov et al. 2008; Ziegler et al. 2008; Blair et al. 2009; Wang et al. 2010, 2013, 2014; Yang and Chan 2015; Zhou et al. 2018.

Character	*G. kuroiwae* group	*G. lichtenfelderi* group	*G. luii* group	*G. yingdeensis* group (3 species)		
	(5 spp.)	(4 spp.)	(7 spp.)	*G. varius* sp. nov.	*G. yingdeensis*	*G. zhelongi*
Scales of upper eyelid one-half the size of scales on the top of head or equal in size	Equal	Equal	Equal or 1/2	Equal	Equal	Equal
Enlarged row of supraorbital tubercles	Absent	Absent or present	Absent or present	Absent	Absent or indistinct	Absent or indistinct
Eyelid fringe scales	<52	43–77	41–67	50–56	46–64	42–53
No. of paravertebral tubercles	Unknown	23–36	27–38	27–29	22–33	28–33
Dorsal tubercle rows at midbody	Unknown	19–22	20–25	21–24	20–25	23–28
Scales around midbody	Unknown	95–140	112–147	101–110	101–116	99–109
Nasal scales surrounding nares	Unknown	8–9	5–9	7–9	7–11	6–8
Internasals	Unknown	1	0–3	1–2	1–3	1–2
Tubercles between orbits	Present or absent	Present or absent	Present or absent	Present	Present	Absent
Claws sheathed by scales	Absent	Present	Present	Present	Present	Present
Lateral scales of claw sheaths	Absent	Long, curved	Long, curved	Short, shell-shaped	Short, shell-shaped	Short, shell-shaped
No. of precloacal pores in males	0	17–46	16–33	10	10–13	9–12
Posterior shape of nuchal loop	Rounded	Protracted or rounded	Protracted	Rounded	Rounded	Rounded
No. of body bands between nuchal loop and the caudal constriction	3 or 4	3 or 4	3, 4 or 5	4	4	4
Dark spotting in body bands	Present or absent	Present or absent	Present or absent	Present or absent	Absent	Absent
Lateral spotting on belly present or absent	Absent	Absent	Present or absent	Present	Present	Present

##### Goniurosaurus yingdeensis

Taxon classificationAnimalia

Wang, Yang & Cui, 2010

27507C44-5AB2-5A81-9A5D-F4F5B093DDA1

**Figure 5.**
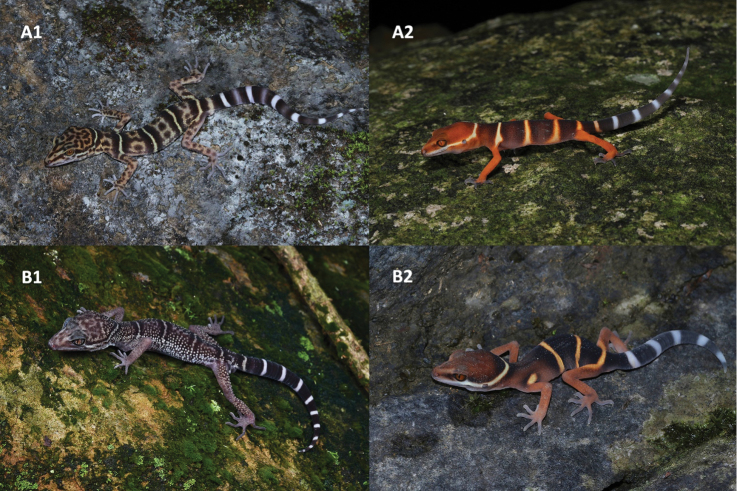
The general aspects of Goniurosaurus yingdeensis and Goniurosaurus zhelongi**A**Goniurosaurus yingdeensis**B**Goniurosaurus zhelongi; (1) adult; (2) juvenile. Photographs by Jian Wang and Shuo Qi.

**Figure 6.**
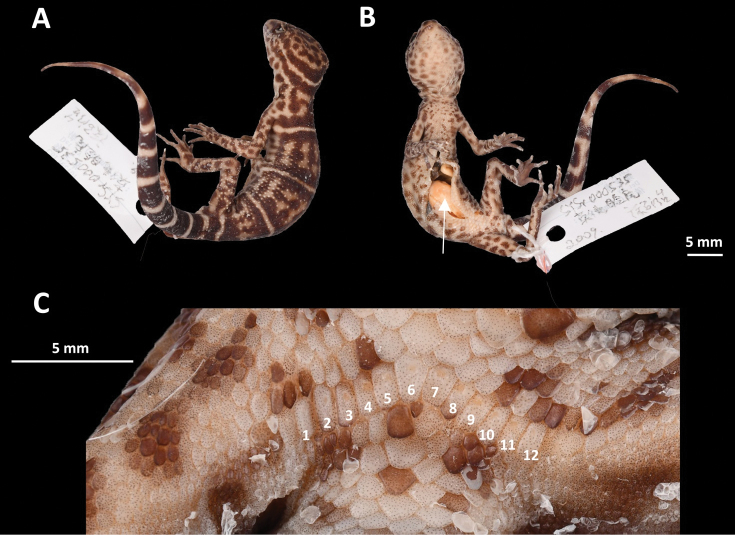
Adult female paratype (SYS r000535) of Goniurosaurus yingdeensis**A** dorsal view **B** ventral view, the white arrow denotes an egg in the fallopian tube **C** close-up of the precloacal region. Photographs by Shuo Qi.

Figs 4A , 5A , 6 , 8B , 9B [English name: Yingde Leopard Gecko] [Chinese formal name: 英德睑虎]

###### Type material.

***Holotype*.**SYS r000504, adult male, collected from Guoshanyao Village, Yingde City, Guangdong Province, China. ***Paratypes*.** Five specimens from the same locality as holotype. Three adult males SYS r000501–0503, an adult female SYS r000535 and a juvenile female SYS r000536. 

###### Additional specimens.

Four adult males (SYS r000788, SYS r000815, SYS r001493, SYS r002115); two adult females (SYS r001271–1272), a subadult female (SYS r000536) and a juvenile female (SYS r000552). All specimens collected from the Shimentai National Nature Reserve.

###### Variation.

 Overall morphology, coloration, and scalation data of the newly discovered populations of G. yingdeensis are in general agreement with the description of the holotype by Wang et al. (2010). Males have 10–13 distinct precloacal pores, whereas precloacal pores are present but indistinct in two adult females (SYS r000535, SYS r001652) and a subadult female (SYS r000536), absent in another two adult females (SYS r001271, SYS r001272); internasal usually numbering two or three, but single in the two females (SYS r001271, SYS r001652). Additional variation in scale counts and measurements are shown in Table 6. For female precloacal pores see Fig. 6. 

**Table 6.** Mensural (mm) and meristic diagnostic characters (minimum/maximum) of specimens of Goniurosaurus yingdeensis. See Materials and methods for abbreviations. * holotype, # paratype.

Morphological character	SYS r000501 #	SYS r000503 #	SYS r000504 *	SYS r000535 #	SYS r000536 #	SYS r000552	SYS r000788	SYS r000815	SYS r001271	SYS r001272	SYS r001493	SYS r001652	SYS r002115
**Sex**	Male	Male	Male	Female	Sub. female	Juv. female	Male	Male	Female	Female	Male	Female	Male
**SVL**	93.1	82.0	91.5	93.8	75.9	67.8	83.1	84.2	87.6	87.3	90.4	86.0	96.3
**TaL**	Regenerated	Regenerated	90.5	88.0	69.9	61.6	Regenerated	Regenerated	Regenerated	Regenerated	Regenerated	Regenerated	90.7
**HL**	24.5	21.7	24.0	23.7	19.0	20.3	24.6	26.3	27.3	26.6	27.3	25.4	29.4
**HW**	16.7	14.5	15.7	17.5	13.1	13.4	14.4	16.5	15.8	15.9	17.2	15.9	17.7
**SE**	9.5	9.0	8.8	9.5	7.8	7.1	9.3	10.0	9.8	9.8	10.1	9.8	10.1
**EE**	9.5	8.4	8.8	9.5	7.6	6.9	8.2	9.0	8.8	8.8	9.3	8.7	9.7
**SPL**	9/8	9/7	9/9	8/9	9/9	10/9	9/9	8/9	11/11	10/9	8/8	8/10	10/9
**IFL**	8/8	8/9	9/9	9/8	8/8	10/10	7/7	9/8	10/9	8/8	9/9	8/8	9/8
**N**	8/8	8/8	8/8	8/8	8/8	8/7	7/7	8/7	11/9	8/8	9/8	8/9	8/9
**IN**	2	3	2	2	3	3	2	3	1	3	2	1	3
**PostIN**	4	4	3	3	5	5	2	4	3	6	4	3	4
**PM**	3	3	4	3	2	3	2	2	4	3	3	3	3
**GP**	7	7	6	7	7	7	5	5	8	7	6	6	6
**CIL**	51/53	56/57	51/49	48/46	53/51	63/64	56/55	57/56	48/46	51/53	58/57	57/56	50/52
**PO**	19/17	17/19	18/16	17/20	16/18	17/18	16/16	19/21	17/16	18/16	17/17	16/17	15/18
**GST**	9–11	9–11	9–11	8–11	8–11	9–12	9–11	8–11	9–12	8–12	8–12	9–11	9–11
**PTL**	28	29	25	25	25	25	32	33	32	33	28	22	26
**DTR**	22	21	21	23	20	22	20	21	23	22	24	21	25
**MB**	111	109	108	115	102	110	113	116	101	106	109	108	114
**PP**	10	13	10	12	10	0	0	13	0	0	12	10	10
**PAT**	2/2	2/2	2/2	2/2	2/2	2/2	2/2	2/2	2/2	2/2	2/2	2/2	2/2

###### Diagnosis.

(1) medium size, measuring 82.0–96.3 mm in SVL in adults; (2) TaL and SVL almost equal in adult with original tail; (3) nasal scales surrounding nares 7–11; (4) internasals 1–3; (5) eyelid fringe scales 46–64; (6) granular scales of the upper eyelids similar in size to those on the top of the head; (7) scales around midbody 101–116; (8) dorsal tubercle rows at midbody 20–25; (9) paravertebral tubercles between limb insertions 22–33; (10) claws sheathed by four scales, two lateral scales short and shell-shaped; (11) axillary pockets deep; (12) precloacal pores 10–13, distinct in males, barely visible or not visible in females; (13) dorsal ground color of head, body, and limbs of adults brown; (14) presence of a thin, cream colored nuchal loop, posteriorly rounded; (15) presence of four thin, cream colored, and immaculate body bands between the nuchal loop and the caudal constriction, edged in black anteriorly and posteriorly; (16) body bands without dark spots; (17) chin, throat, thorax, and ventral surfaces of limbs white, dark brown spots present, ventral surfaces of body dull white, interspersed with dark brown scales, dark brown lateral spots on belly; (18) iris gray, becoming orange near pupil.

###### Distribution.

Goniurosaurus yingdeensis is currently only known from the Yingde City, Guangdong Province, China. 

##### Goniurosaurus zhelongi

Taxon classificationAnimalia

Wang, Jin, Li & Grismer, 2014

C49C7A04-4BB7-5FEE-A596-4C6ED61837FE

Figs 5B , 8C , 9C [English name: Zhe-Long’s Leopard Gecko] [Chinese formal name: 蒲氏睑虎]

###### Type material.

***Holotype*.**SYS r000770, adult male, collected from the Shimentai National Nature Reserve, Yingde City, Guangdong Province, China. ***Paratypes*.** Four specimens, bearing the same data as the holotype. Three adult females SYS r000551, SYS r000765–0766 and one adult male SYS r000816.

###### Additional specimens.

Two adult males (SYS r001491, SYS r001492) and an adult female (SYS r002108). All specimens collected from the Shimentai National Nature Reserve.

###### Variation.

 Overall morphology, coloration, and scalation data of the newly discovered populations of *G. zhelongi* are in general agreement with the description of the holotype by Wang et al. (2014). Precloacal pores usually nine in males, 12 in an adult male (SYS r001491) and absent in female; internasal single or two. Additional variation in scale counts and measurements are shown in Table 7.

**Table 7.** Mensural (mm) and meristic diagnostic characters (minimum/maximum) of specimens of *Goniurosaurus zhelongi*. See Materials and methods for abbreviations. * holotype, # paratype.

Morphological character	SYS r000551 #	SYS r000765 #	SYS r000766 #	SYS r000770 *	SYS r000816 #	SYS r001491	SYS r001492	SYS r002108
**Sex**	Female	Female	Female	Male	Male	Male	Male	Female
**SVL**	91.5	93.4	91.6	86.0	88.1	90.8	87.1	87.9
**TaL**	Regenerated	79.6	Regenerated	Regenerated	Regenerated	Regenerated	77.8	80.0
**HL**	24.4	23.4	23.9	22.4	22.8	25.5	23.3	24.5
**HW**	15.8	15.9	16.1	15.6	15.7	17.1	14.8	16.0
**SE**	9.7	9.5	9.5	8.9	9.1	9.5	8.3	9.2
**EE**	9.8	9.9	9.9	9.5	9.6	9.2	7.8	8.5
**SPL**	8/8	7/7	8/8	9/8	9/8	9/8	9/9	10/8
**IFL**	7/7	6/7	7/7	7/7	7/7	9/9	8/8	8/8
**N**	7/7	7/8	6/6	8/8	7/7	8/7	7/8	7/7
**IN**	1	1	1	2	1	2	2	1
**PostIN**	3	3	3	3	3	3	3	3
**PM**	6	4	6	4	5	5	5	4
**GP**	9	7	8	8	8	9	7	9
**CIL**	47/48	47/48	48/45	52/48	49/51	47/46	43/42	52/53
**PO**	14/14	13/15	15/15	14/15	13/14	16/16	14/13	16/17
**GST**	10–12	9–11	11–12	9–12	10–12	9–11	10–12	9–11
**PTL**	33	28	29	29	28	28	28	29
**DTR**	26	26	27	28	28	23	24	23
**MB**	108	105	109	108	109	99	105	103
**PP**	0	0	0	9	9	12	9	0
**PAT**	2/2	2/2	2/2	2/2	2/2	2/2	2/2	2/2

###### Diagnosis.

(1) medium size, measuring 86.0–93.4 mm in SVL in adults; (2) TaL 0.85 times as long as SVL; (3) nasal scales surrounding nares 6–8; (4) internasal one or two; (5) eyelid fringe scales 42–53; (6) granular scales of the upper eyelids similar in size to those on the top of the head; (7) scales around midbody 99–109; (8) dorsal tubercle rows at midbody 23–28; (9) paravertebral tubercles between limb insertions 28–33; (10) claws sheathed by four scales, two lateral scales short and shell-shaped; (11) axillary pockets deep; (12) 9–12 precloacal pores in males and absent in females; (13) dorsal ground color of head, body, and limbs of adults brownish-black; (14) a thin, cream colored, posteriorly rounded nuchal loop; (15) four thin, cream colored, and immaculate body bands between the nuchal loop and the caudal constriction, edged in black anteriorly and posteriorly; (16) body bands without dark spots; (17) chin, throat, thorax, and ventral surfaces of body white, tinged brownish, with dark brown lateral spots; (18) iris gray-white, tinged with orange.

###### Distribution.

*Goniurosaurus zhelongi* is currently only known from the Shimentai National Nature Reserve, Yingde City, Guangdong Province, China.

##### Goniurosaurus varius sp. nov.

Taxon classificationAnimalia

9CEB8640-C35C-5964-B7F7-E3F7A223792D

http://zoobank.org/E28DD1FB-9EDD-4E6A-ACAE-5F831BC310BD

**Figure 7.**
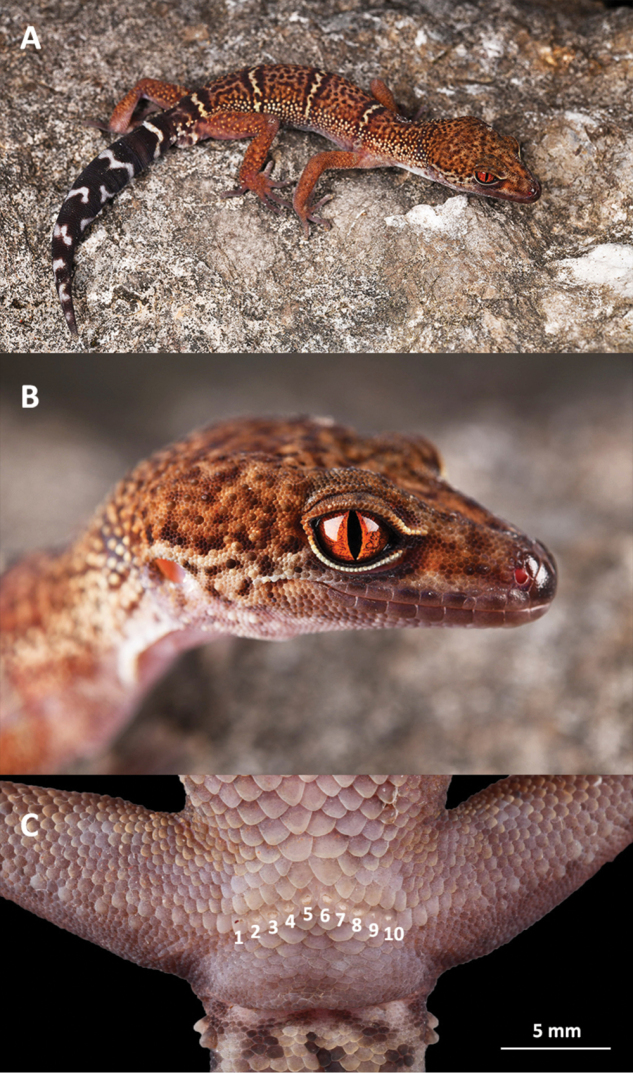
**A** Dorsolateral view of the adult male holotype SYS r002333 of *Goniurosaurus varius* sp. nov. in life **B** scalation and coloration characters of the head of the adult male holotype SYS r002333 of *Goniurosaurus varius* sp. nov. **C** ten precloacal pores in adult male holotype SYS r002333. Photographs by Shuo Qi.

**Figure 8.**
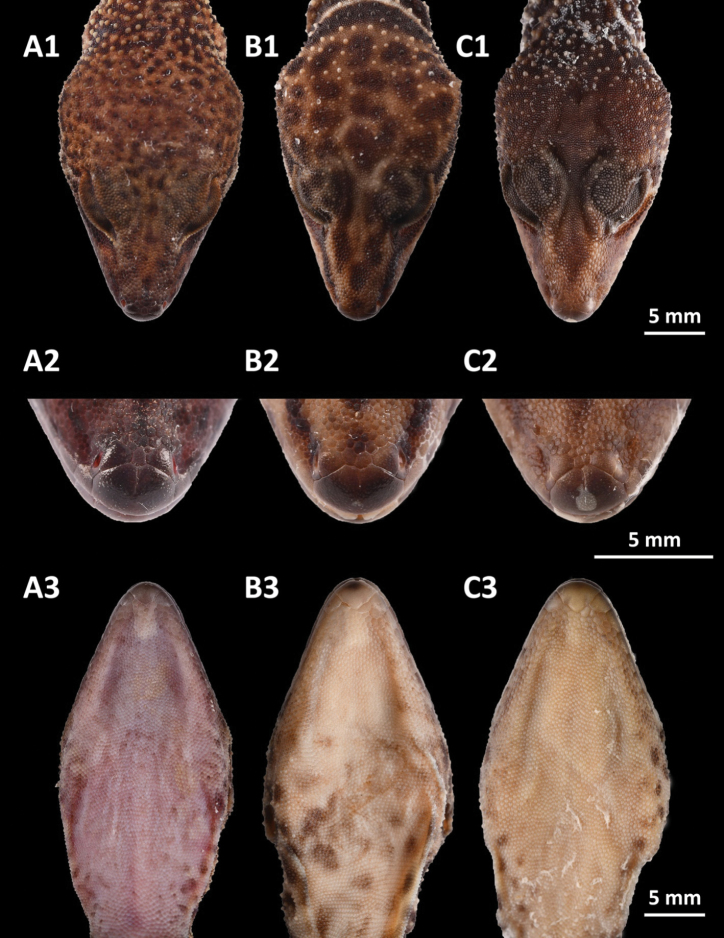
Comparisons of head morphological characteristics with two closely related congeners **A***Goniurosaurus varius* sp. nov. (holotype, SYS r002333) **B***Goniurosaurus yingdeensis* (SYS r001943) **C***Goniurosaurus zhelongi* (holotype, SYS r000770) **1** dorsal view **2** close-up of dorsal snout **3** Ventral view. Photographs by Shuo Qi.

**Figure 9.**
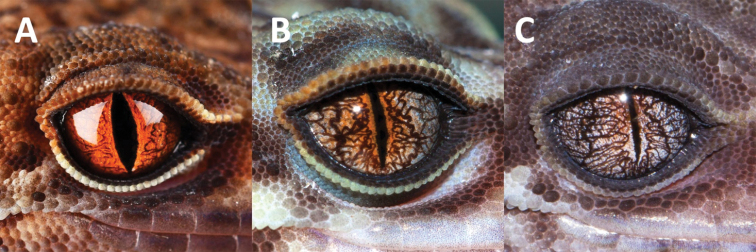
Comparisons of iris color with two closely related congeners **A***Goniurosaurus varius* sp. nov. (holotype, SYS r002333) **B***Goniurosaurus yingdeensis* (holotype SYSr000504) **C***Goniurosaurus zhelongi* (holotype, SYS r000770). Photographs by Shuo Qi and Ying-Yong Wang.

**Figure 10.**
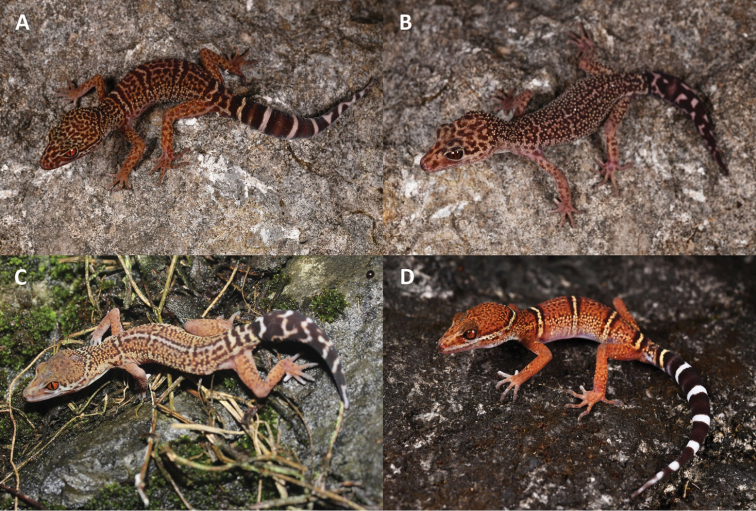
Differently patterned morphs of adult and juvenile coloration in *Goniurosaurus varius* sp. nov. **A** cross-banded morph **B** mottled morph **C** striped morph **D** juvenile coloration. Photographs by Shuo Qi and Peng Cen.

Figs 7 , 8A , 9A , 10 [English name: Nanling Leopard Gecko] [Chinese formal name: 南岭睑虎]

###### Type material.

***Holotype*.**SYS r002333, adult male (Fig. 7), collected by Liang Zhang on 20 September 2019 from Nanling National Nature Reserve (ca 560 m a.s.l.), Chengjia Yao Ethnic Township, Yangshan County, Guangdong Province, China. ***Paratypes*.** One adult male (SYS r002331) and three adult females (SYS r002330, SYS r002362–2363), collected by Zhi-Ren Zhang, Yu Zhang, and Peng Cen on 6 August 2018, from Nanling National Nature Reserve, Chengjia Yao Ethnic Township at elevations between 180 and 560 m.

###### Additional specimens.

Five individuals from the Nanling National Nature Reserve, Yangshan County, including a road-killed adult (SYS r002357), and four captured/released individuals (two juveniles, one adult male, and one adult female). All released individuals were photographed and measured for morphological examination. The tips of the tails were removed for future molecular analyses (SYS r002355, 2358, 2359, 2360), but not used these in current phylogenetic analysis.

###### Etymology.

 The specific name *varius* means varied or diverse in Latin and refers to its variable dorsal color pattern. As the type locality locates within the Nanling National Nature Reserve, we suggest the common name as “Nanling Leopard Gecko”.

###### Diagnosis.

*Goniurosaurus varius* sp. nov. can be distinguished from all other congeners by the combination of the following characters: (1) adult body size moderate, measuring 81.5–86.3 mm in SVL; (2) nasal scales surrounding nares 7–9; (4) internasal usually single, rarely two; (5) eyelid fringe scales 50–56; (6) granular scales of the upper eyelids similar in size to those on the top of the head; (7) scales around midbody 101–110; (8) dorsal tubercle rows at midbody 21–24; (9) paravertebral tubercles between limb insertions 27–29; (10) claws sheathed by four scales, dorsal scale small, two lateral scales short and shell-shaped; (11) axillary pockets deep; (12) presence of ten precloacal pores in males, and absent in females; (13) dorsal ground color of head, body, and limbs in adults reddish brown, mottled with varied spots and stripes; (14) nuchal loop usually incomplete, if complete, posteriorly rounded; (15) presence of four thin dorsal body bands with dark spots, bordered with black anteriorly and posteriorly, sometime last two bands indistinct; (16) usually presence of a longitudinal light colored vertebral stripe on the trunk of body; (17) light pink beneath, with dark brown lateral spots; (18) iris orange-red.

###### Comparisons.

*Goniurosaurus varius* sp. nov. is most similar to *G. yingdeensis* and *G. zhelongi*, two closely related species from north Guangdong Province, but it differs from them by following characters: paravertebral tubercles between limb insertions 27–29 (25–26 in *G. yingdeensis*, 28–33 in *G. zhelongi*); dorsal tubercle rows at midbody 21–24 (vs. 25–27 in *G. yingdeensis*, 23–28 in *G. zhelongi*); trunk of body usually with a longitudinal light colored vertebral stripe (vs. absent in *G. yingdeensis* and *G. zhelongi*); nuchal loop and body bands with black spots (vs. without black spots in *G. yingdeensis* and *G. zhelongi*); iris orange-red (vs. iris gray, becoming orange near pupil in *G. yingdeensis*, iris gray-white, tinged with orange in *G. zhelongi*). Additional comparisons of morphological characteristics with *G. yingdeensis* and *G. zhelongi* are provided in Figures 8, 9.

###### Description of holotype.

An adult male with regenerated tail; SVL 84.7 mm; HL 22.7 mm; HW 16.0 mm; SE 9.1 mm; EE 13.0 mm; SVL:HL 3.7; HL:HW 1.4; SE:EE 0.7. Head triangular, wider than neck, covered with granular scales, densely interspersed with tubercles in the temporal and occipital regions; area between orbits uniformly covered by small granular scales; supraorbital tubercles with almost uniform size; scales of rostrum slightly larger than those in between orbits; rostral convex and hemi-elliptic, 1.3 times as broad as high, middorsal portion of rostral partially sutured dorsomedially, bordered laterally by first supralabial and prenasal, dorsolaterally by supranasal, dorsally by one internasal; external nares oval, surrounded by 7/8 nasals each, anteriorly by prenasal and supranasal, dorsally by supranasal and a granular scale, posteriorly by 5/5 smaller granular scales, and ventrally by the prenasal; prenasal with long recurved ventral portion; supranasals large, separated by one internasals; supralabials rectangular, 8/10; preorbital scales 15/15; eyes relatively large, pupils vertical; eyelid fringe scales 50/52; outer surface of upper eyelid composed of granular scales of about the same size of those on top of head; external auditory meatus circular, tympanum deeply recessed; mental triangular, bordered laterally by first infralabial and posteriorly by three postmentals; infralabials rectangular, 9/9; gular scales juxtaposed uniform granular, abruptly into flat juxtaposed pectoral scales, and grading posteriorly imbricated larger ventral scales. Tongue with a small notch at tip. Crowns of teeth expanded, occlusal margins bearing multiple ridges.

Dorsal surface of neck and body covered with uniform granular scales, interspersed with densely sharply pointed conical tubercles; scales around midbody 105; dorsal tubercle rows at midbody 24; vertebral row of scales indistinct; paravertebral tubercles between limb insertions 27; dorsal body tubercles surrounded by 9–11 granular scales; dorsal scales grading ventrally into larger flattened imbricate ventral scales; ten precloacal pores in a transverse series; postcloacal region greatly swollen, covered with imbricate flattened scales, containing 2/2 postcloacal tubercles laterally at the level of the vent.

Regenerated tail, short, thin at base, gradually thickening posteriorly, and gradually thinning into an obtuse tip; dorsal scales in regenerated portion of tail flattened, subimbricate, arranged in more or less regular transverse rows; subcaudal scales flattened, smooth, subimbricate, slightly larger than dorsal caudal scales.

Limbs relatively long and slender; dorsal surface covered with granular scales, densely interspersed with tubercles; ventral surface covered by flat scales, juxtaposed, subimbricate or imbricate; dorsal surface of pes and manus covered with granular scales, interspersed with several conical tubercles on top of pes, lacking tubercles on top of manus; hind limbs slightly larger than forelimbs; ventral surfaces of pes and manus covered with large granular scales; axillary pockets deep; subdigital lamellae wide, 7/7 on Finger I, 12/12 on Finger II, 15/16 on Finger III, 17/15 on Finger IV, 13/13 on Finger V, 8/8 on Toe I, 13/ 13 on Toe II, 17 / 17 on Toe III, 21 / 18 on Toe IV, and 18 / 15 on Toe V; fingers laterally compressed, relative finger lengths I < V<II < III ≤ IV; toes laterally compressed, third toe nearly as long as the fourth toe, relative toe length I < II < V ≤ III < IV; base of claws sheathed by four scales, two lateral scales of claw short shell-shaped.

###### Coloration in life.

Dorsal ground color of head, neck, body, and limbs reddish brown, mottled with indistinct faint dark brown-colored markings, scattered with densely light yellow tubercles and a few dark brown and reddish brown tubercles; nuchal loop incomplete, just from posterior corner of eyes to the temporal region, dirty yellow; four narrow body bands between the nuchal loop and the caudal constriction, fourth band inserting into the dorsal thigh, bands dirty yellow, with dark spots, edged in dark-brown anteriorly and posteriorly; a longitudinal light colored vertebral stripe between third and fourth bands; supralabials and infralabials grayish brown; pupils vertical and black; iris orange-red; dorsal surface of limbs deep reddish brown with dirty yellow tubercles and indistinct dark spots; chin, throat, thorax, and ventral surfaces of body pink, tinged brownish, with dark-brown lateral spots; ventral surface of limbs pink, tinged brownish, with dark-brown spots; digits gray; ground color of the regenerated tail dark-brown, one original white band on the bases of tail, followed by irregularly shaped white markings. The body color becomes darker after capture.

###### Coloration in preservative.

Dorsal ground color of head, body, and limbs black; ventral surface faded to grayish white; all darker spots and bands on the dorsal surface blurred.

###### Coloration in juvenile.

Dorsal ground color of head, neck, body, and limbs dark-orange, mottled with indistinct faint dark-brown-colored markings, scattered with dense light yellow tubercles and a few dark-brown tubercles; nuchal loop from posterior corner of the mouth to the back of head, light yellow; four narrow body bands between the nuchal loop and the caudal constriction, fourth band inserting into the dorsal thigh, band color light yellow with dark spots, edged in dark-brown anteriorly and posteriorly (but not laterally); supralabials and infralabials grayish brown; pupils vertical and black; iris orange-red; dorsal surface of limbs dark orange with orange tubercles and indistinct dark spots; chin, throat, thorax, and ventral surfaces of body pink; ventral surface of limbs pink with dark-brown spots; digits gray; tail black-grey bearing white caudal bands encircling tail.

###### Variations.

Measurements of type series specimens are shown in Table 8. Three paratypes have more complete and distinct nuchal loops than holotype, but SYS r002363 has only half a nuchal loop from the posterior corner of the right eye to the back of head. SYS r002330 has vertebral stripe extending from the posterior edge of the second body band to the anterior edge of third body band. SYS r002331 and SYS r002363 have large dark dorsal blotches on the head and the body band margin are broader than those in the holotype. Also, SYS r002363 has immaculate body bands. An additional female specimen (Fig. 10B) shows a more mottled dorsal pattern than all other types and its bands are mingled with irregular patterns on the body. SYS r002362 (Fig. 10C) has smaller dorsal blotches making it appear almost as if it has a reticulated dorsal pattern and its bands are greatly obscured, it has a distinct white vertebral stripe from the posterior edge of the first body band extending to the anterior edge of the last body band.

**Table 8.** Mensural (mm) and meristic diagnostic characters (minimum/maximum) of type series of *Goniurosaurus varius* sp. nov. See Materials and methods for abbreviations. * holotype, # paratype.

Morphological character	SYS r002330 #	SYS r002331 #	SYS r002333 *	SYS r002362 #	SYS r002363 #
**Sex**	female	male	male	female	female
**SVL**	86.3	84.9	84.7	81.5	85.7
**TaL**	Regenerated	Regenerated	Regenerated	Regenerated	Regenerated
**HL**	22.3	22.9	22.7	21.5	23.5
**HW**	14.7	15.0	16.0	14.5	15.6
**SE**	8.7	8.8	9.1	8.7	10.0
**EE**	12.4	14.3	13.0	12.8	13.2
**SPL**	8/8	9/9	8/10	9/8	7/7
**IFL**	8/8	9/9	9/9	9/8	9/9
**N**	7/7	8/7	7/8	7/8	7/9
**IN**	1	2	1	2	1
**PostIN**	4	4	3	4	4
**PM**	3	3	3	3	4
**GP**	7	8	8	6	8
**CIL**	52/54	54/56	51/53	51/50	53/55
**PO**	11/12	14/15	15/15	14/15	16/16
**GST**	9–11	9–11	9–11	8–11	9/12
**PTL**	28	28	27	28	29
**DTR**	24	24	24	21	24
**MB**	104	101	105	107	110
**PP**	Absent	Injured, unable to count	10	Absent	Absent
**PAT**	2	2	2	2	2

###### Distribution and ecology.

**Figure 11.**
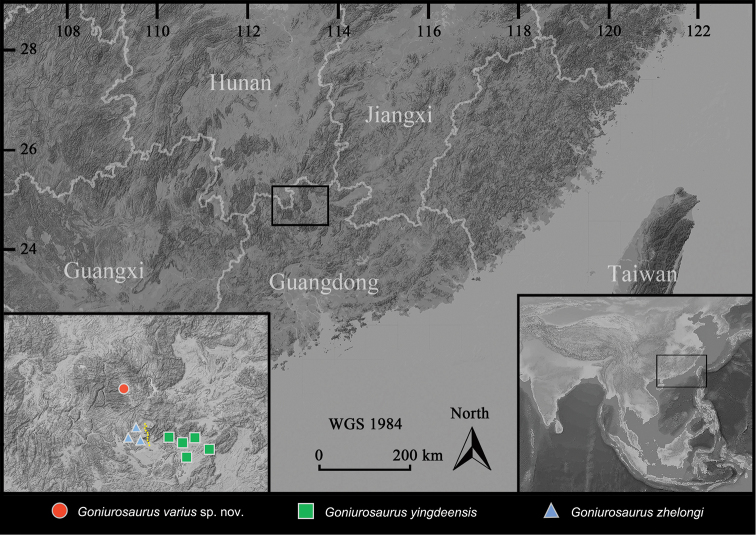
Geographic distribution of three species of *Goniurosaurus yingdeensis* group, the background depicts altitude in the southern China and the provinces of the region (darker shades indicating higher altitudes). The inset on the bottom left shows the detailed distribution, red circle indicates the collecting locality of the *Goniurosaurus varius* sp. nov., green squares and blue triangles indicate that known distributions of *G. yingdeensis* and *G. zhelongi*, respectively. The yellow dotted line indicates the Ruyuan Canyon. The bottom right inset shows the location of the main map in a regional context. Geographical basic map source from Google Maps.

*Goniurosaurus varius* sp. nov. is currently known only from the karst environment of the Nanling National Nature Reserve, northern Guangdong Province, China (Figure 11). All individuals were found in crevices of limestone near villages, farmlands, or country lanes at elevations between 180 and 560 m at night after 21:00 hrs.

## Discussion

Our continued herpetological surveys coupled with extensive sampling in Guangdong Province, China in the past decades have resulted in discovery of three new species of *Goniurosaurus* from two localities, which all belong to the *G. yingdeensis* group. Topographically, rivers and a canyon form a series of geographic barriers that might lead to the isolations of members of *G. yingdeensis* group. Among them, *G. yingdeensis* is distributed in the lower hill areas on the east side of the Ruyuan Canyon, *G. zhelongi* was found on the west side of canyon. Moreover, microhabitat selection might also play an important role in species differentiation. Nearly all of *G. varius* individuals were found in karst topography but *G. yingdeensis* and *G. zhelongi* were also found in granitic landforms. This suggests they may be saxicolous generalist as opposed to a microhabitat specialist. Future phylogeographic and habitat selection studies are needed to gain a better understanding of their evolutionary history.

As the development of integrated taxonomy, to combine the morphological comparisons and phylogenetic relationships, has become an important and necessary work. In the present study, we propose the morphological definition of the *Goniurosaurus yingdeensis* group, which can be significantly distinguished from all other congeners, consistent with their distinct divergences in phylogeny. Nevertheless, it is worth noting that the species *G. bawanglingensis* and *G. zhoui* can be assigned to the *G. luii* group according to previous morphological diagnoses (Grismer et al. 2002; Zhou et al. 2018), while they were clustered within *G. lichtenfelderi* group in phylogeny based on two mitochondrial and two nuclear genetic segments (Liang et al. 2018). Hence, further comprehensive work with detailed morphological examinations and more genetic data is asked for, to clarify these incongruences or revise the morphological definitions of the *G. luii* group and the *G. lichtenfelderi* group.

## Supplementary Material

XML Treatment for Goniurosaurus yingdeensis

XML Treatment for Goniurosaurus zhelongi

XML Treatment for Goniurosaurus varius

## Appendix 1

**Examined specimens**

*Goniurosaurus bawanglingensis* (*N* = 3): China: Hainan Province: Bawangling National Nature Reserve: SYS r001075, 1670, 2162.

*Goniurosaurus hainanensis* (*N* = 2): China: Hainan Province: Jianfengling National Forest Park: SYS r000349; Baoting County: SYS r001270.

*Goniurosaurus liboensis* (*N* = 3): China: Guizhou Province: Libo County: Maolan National Nature Reserve: SYS r000217, 854, 855.

*Goniurosaurus luii* (*N* = 4): China: Guangxi Zhuang Autonomous Region: Jingxi City: SYS r000255, 256, 859, 860.

*Goniurosaurus yingdeensis* (*N* = 10): China: Guangdong Province: Yingde City: SYS r000501, 503, 504, 535, 536, 550, 1271, 1272, 1493, 2115.

*Goniurosaurus zhelongi* (*N* = 5): China: Guangdong Province: Yingde City: SYS r000816, 1491, 1492, 2011, 2108.

*Goniurosaurus zhoui* (*N* = 2): China: Hainan Province: SYS r002213, 2214.
